# Eyes shut homolog is required for maintaining the ciliary pocket and survival of photoreceptors in zebrafish

**DOI:** 10.1242/bio.021584

**Published:** 2016-10-13

**Authors:** Miao Yu, Yu Liu, Jing Li, Brianna N. Natale, Shuqin Cao, Dongliang Wang, Jeffrey D. Amack, Huaiyu Hu

**Affiliations:** 1 Center for Vision Research, Departments of Ophthalmology and Neuroscience and Physiology, SUNY Upstate Medical University, Syracuse, NY 13210, USA; 2 Department of Public Health and Preventive Medicine, SUNY Upstate Medical University, Syracuse, NY 13210, USA; 3 Department of Cell and Developmental Biology, SUNY Upstate Medical University, Syracuse, NY 13210, USA

**Keywords:** Connecting cilia, Degeneration, Extracellular matrix, Photoreceptors, Retinal degeneration, Retinitis pigmentosa

## Abstract

Mutations in the extracellular matrix protein eyes shut homolog (EYS) cause photoreceptor degeneration in patients with retinitis pigmentosa 25 (RP25). Functions of EYS remain poorly understood, due in part to the lack of an EYS gene in mouse. We investigated the localization of vertebrate EYS proteins and engineered loss-of-function alleles in zebrafish. Immunostaining indicated that EYS localized near the connecting cilium/transition zone in photoreceptors. EYS also strongly localized to the cone outer segments and weakly to the rod outer segments and cone terminals in primate retinas. Analysis of mutant EYS zebrafish revealed disruption of the ciliary pocket in cone photoreceptors, indicating that EYS is required for maintaining the integrity of the ciliary pocket lumen. Mutant zebrafish exhibited progressive loss of cone and rod photoreceptors. Our results indicate that EYS protein localization is species-dependent and that EYS is required for maintaining ciliary pocket morphology and survival of photoreceptors in zebrafish.

## Abstract (precis)

**Summary:** The extracellular matrix protein Eyes shut homolog is required for maintaining the integrity of the ciliary pocket and survival of photoreceptors in zebrafish.

## INTRODUCTION

Retinitis pigmentosa (OMIM #268000) is a heterogeneous group of genetic diseases exhibiting progressive retinal degeneration due to loss of photoreceptors. It has a worldwide prevalence of 1/3000 to 1/7000. Clinical manifestations of photoreceptor degeneration are highly variable and include night blindness, retinal pigmentation, loss of peripheral vision, and loss of central vision. Depending on which photoreceptor type is lost earlier, photoreceptor degeneration can be characterized as rod-cone or cone-rod dystrophies.

The autosomal recessive retinitis pigmentosa 25 (RP25, OMIM #612424) is caused by abnormal EYS (Abd El-Aziz et al., 2008; Collin et al., 2008), a secreted extracellular matrix protein, in several populations worldwide (Abd El-Aziz et al., 2008, 2010; Audo et al., 2010; Bandah-Rozenfeld et al., 2010; Barragán et al., 2010; Chen et al., 2015; Collin et al., 2008; Di et al., 2016; Hosono et al., 2012; Littink et al., 2010b). Mutations in EYS account for 5-16% of all autosomal recessive cases in Europe (Audo et al., 2010; Barragán et al., 2010; Littink et al., 2010b) and the most prevalent form of inherited retinal dystrophy in Japan (Arai et al., 2015; Iwanami et al., 2012). Loss of photoreceptors has been confirmed by histopathological evaluation of the patients' retina (Bonilha et al., 2015). The human EYS protein has 3165 amino acid residues with multiple EGF and laminin G domains. Most presumed pathogenic mutations are frameshift or nonsense mutations that delete one or more laminin G domains and one or more EGF domains; however, missense mutations that occur in laminin G domains or EGF domains have also been discovered throughout the polypeptide chain (Abd El-Aziz et al., 2008, 2010; Audo et al., 2010; Bandah-Rozenfeld et al., 2010; Barragán et al., 2010; Collin et al., 2008; Hosono et al., 2012; Littink et al., 2010b). Although most patients with EYS mutations are diagnosed as having rod-cone dystrophies, a cone-rod pattern of photoreceptor loss has also been reported (Collin et al., 2008; Katagiri et al., 2014; Littink et al., 2010a).

The *Drosophila* homolog of eyes shut (EYS), also known as spacemaker or spam, is located in inter-rhabdomeral spaces of the compound eye (Husain et al., 2006; Zelhof et al., 2006). It is essential for the creation of the inter-rhabdomeral space because spacemaker mutations result in the collapse of the inter-rhabdomeral space. O-glucosyl glycosylation of several EGF domains of eyes shut is essential because mutation of *Drosophila* glucosyl glycosylation causes a significant reduction in eyes shut protein levels and results in closure of the inter-rhabdomeral space (Haltom et al., 2014). *Drosophila* eyes shut is also expressed at the extracellular space of mechanosensory organs where it provides mechanical support to the sensory cell (Cook et al., 2008; Husain et al., 2006). In vertebrates, EYS proteins are thought to be specifically expressed by the photoreceptors; immunolocalization studies have found the porcine EYS at the outer segment of rod photoreceptors (Abd El-Aziz et al., 2008).

Despite the essential roles for *Drosophila* eyes shut protein in ommatidium development and human EYS in photoreceptor survival, the genomes of the mouse and several other mammalian clades lack a functional EYS locus (Abd El-Aziz et al., 2008). As a result, EYS is poorly characterized in the vertebrate retina. Thus, we have evaluated the distribution of EYS protein in the retinas of several species including the zebrafish, monkey, and human. For functional studies of EYS in vertebrate retina, we generated EYS-deficient zebrafish by gene editing with CRISPR/Cas9. Our results indicate that EYS protein is highly concentrated near the connecting cilium/transition zone (CC/TZ) of photoreceptors and also highly concentrated in the outer segment of primate cones. Analysis of EYS-deficient zebrafish shows that EYS is required for the maintenance of the ciliary pocket and survival of cone photoreceptors as well as survival of rod photoreceptors. These results provide new insight into the functions of EYS and the cellular mechanisms underlying retinal degeneration in RP25 patients with EYS mutations. Mutant EYS zebrafish provide the first vertebrate model of RP25 that may aid in therapeutic discovery.

## RESULTS

### EYS protein is located near connecting cilium/transition zone (CC/TZ) in zebrafish retina

**Fig. 1.**
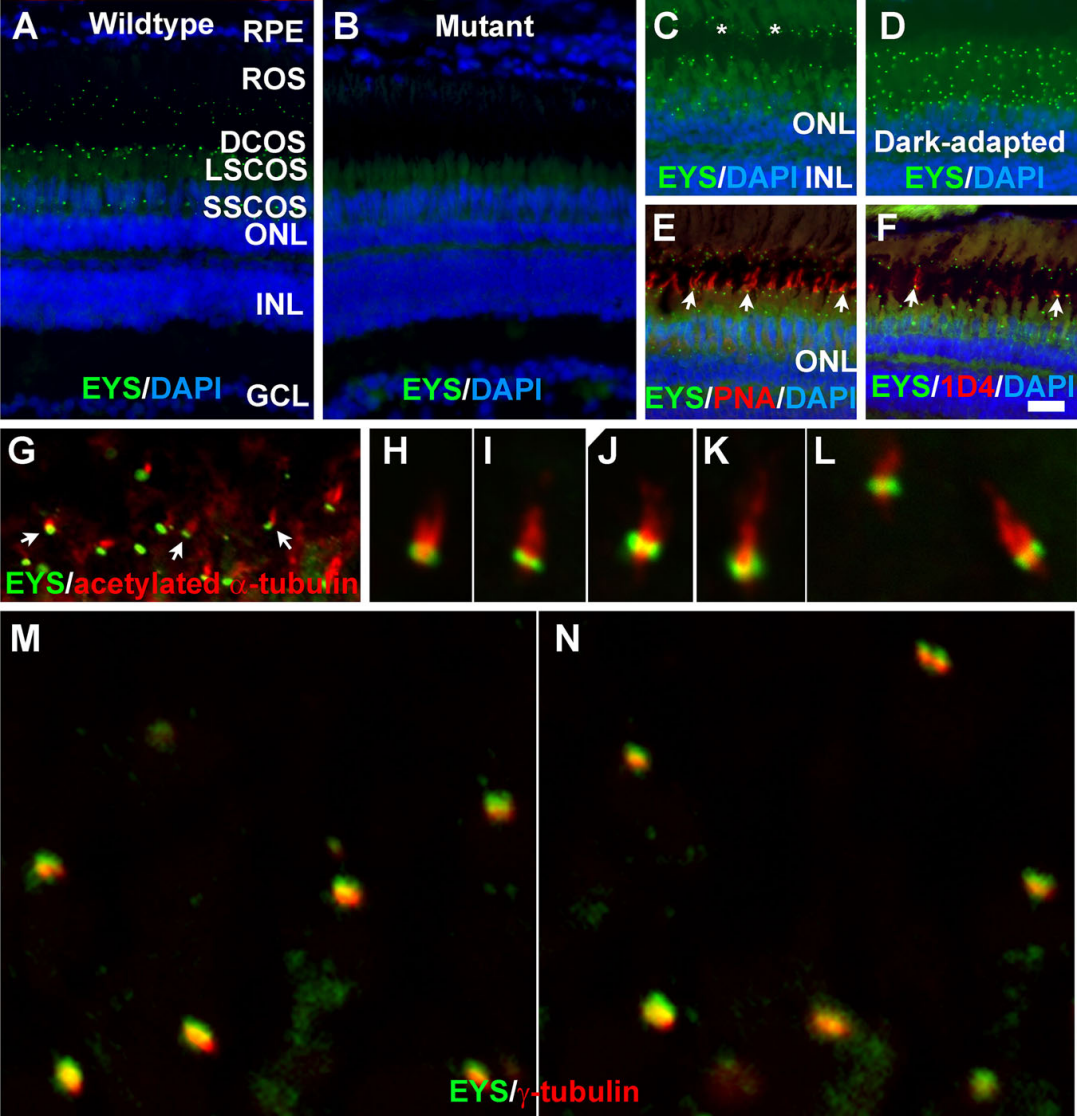
**Zebrafish EYS protein was concentrated near the connecting cilium/transition zone.** Zebrafish eye sections were immunostained with EYS antibody and PNA, and 1D4 antibody and antibodies against acetylated α-tubulin and γ-tubulin. The sections were counter stained with DAPI to show nuclei. (A) EYS antibody staining (green fluorescence) of wild-type retina (*n*=10). EYS immunoreactivity exhibited as a punctate pattern between the RPE and ONL. (B) EYS immunostaining of EYS-deficient retina (*n*=10). Note the absence of punctate fluorescence. (C,D) EYS immunostaining of light- and dark-adapted retinas. Note that some of the EYS punctate were located at the basal end of autofluorescence of rod outer segment (asterisks). The domain of EYS punctate shrank upon dark adaptation. (E,F) Double staining of EYS antibody with PNA and 1D4 antibody, respectively. Note that some of the EYS punctate were located on the basal end of the PNA-positive cone outer segment and 1D4-positive outer segment of long double cones (arrows). (G-L) Double staining of EYS (green) and acetylated α-tubulin (red). Note that EYS immunoreactivity is located on the basal end of acetylated α-tubulin. Arrows in G indicate examples at low magnification. (M-N) Double staining of EYS (green) and γ-tubulin (red) antibodies. Note that EYS is apposed to but apical of γ-tubulin. Scale bar in F: 20 µm for A-F. DCOS, double cone outer segment; GCL, ganglion cell layer; INL, inner nuclear layer; LSCOS, long single cone outer segment; ONL, outer nuclear layer; ROS, rod outer segment; RPE, retinal pigment epithelium; SSCOS, short single cone outer segment.

To determine the distribution of the EYS protein in the zebrafish retina, we carried out immunofluorescence staining with an EYS antibody (Fig. 1). In two-month old retinas, a punctate pattern of strong immunoreactivity was found between the retinal pigment epithelium (RPE) and outer nuclear layer (ONL) (Fig. 1A, green fluorescence). In EYS-mutant retinas, EYS immunoreactivity was completely abolished (Fig. 1B), demonstrating ablation of EYS protein in mutants and confirming specificity of the EYS expression pattern in wild-type animals. EYS-immunoreactive puncta appeared to be located on the basal end of rod outer segments (asterisks in Fig. 1C) and cone outer segments based on autofluorescence (Fig. 1C). The domain of EYS immunoreactive puncta was reduced under dark-adapted conditions (Fig. 1D). Double staining with peanut agglutinin (PNA) (Fig. 1E, arrows) indicated that some EYS immunoreactive puncta were located on the basal end of PNA-positive outer segments. We also carried out double immunofluorescence staining with an EYS antibody and the 1D4 antibody that recognizes outer segments of long double cone in zebrafish (Yin et al., 2012). Some of the punctate EYS immunoreactivity was apposed to and basal to 1D4 immunoreactivity (Fig. 1F, arrows). These results suggested that EYS protein might be located near the CC/TZ.

To determine whether EYS is located near the CC/TZ, we carried out double immunofluorescence staining of the EYS antibody with antibodies against acetylated α-tubulin (axoneme marker) and γ-tubulin (basal body marker). EYS immunoreactive puncta were located on the basal end of acetylated α-tubulin immunoreactivity (Fig. 1G-L) but apical to γ-tubulin (Fig. 1M-N). The fluorescence pattern of EYS at the basal end of acetylated α-tubulin and at the apical side of γ-tubulin suggests that EYS is located near the CC/TZ.

### EYS is also located in the outer segment of primate cone photoreceptors in addition to near the CC/TZ

**Fig. 2.**
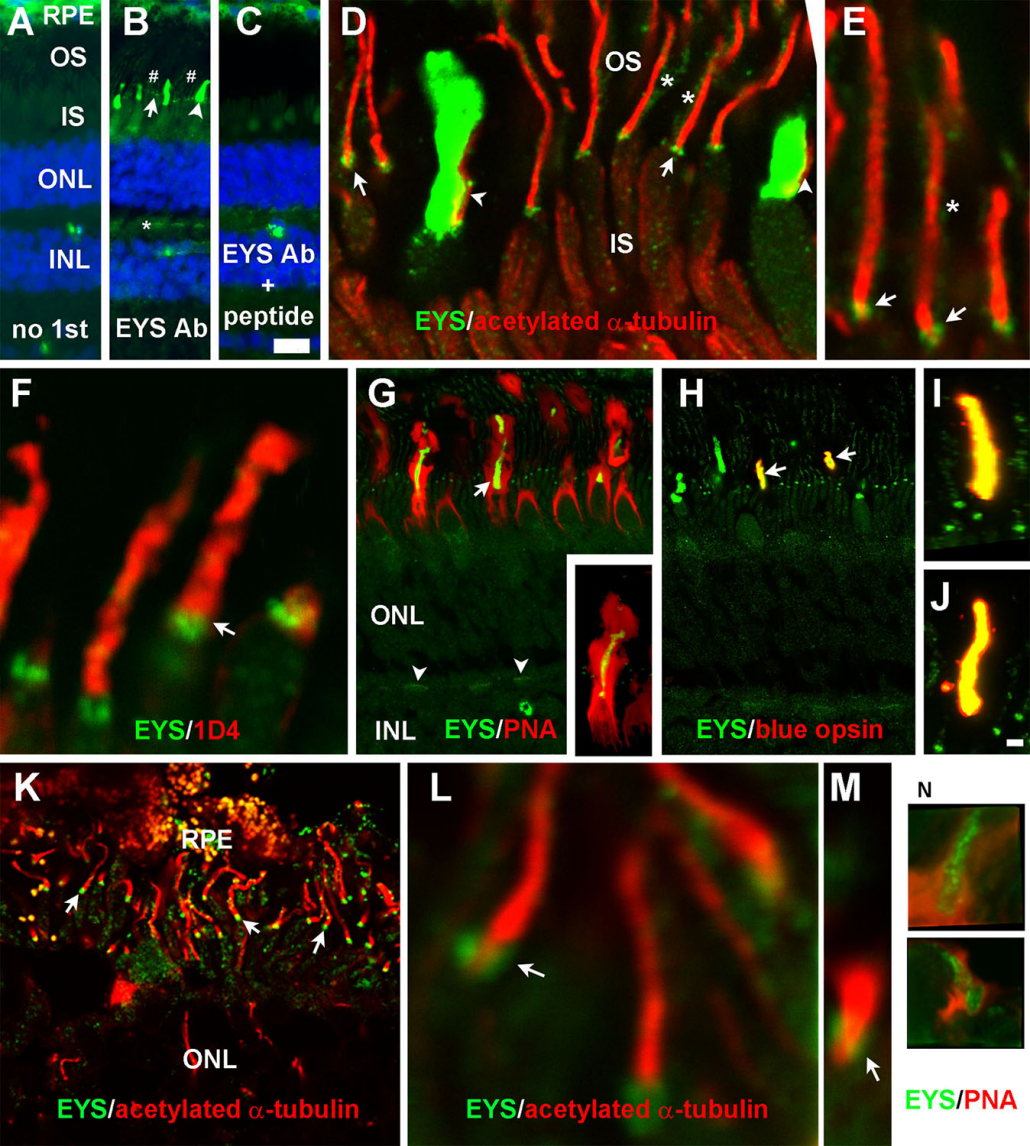
**EYS protein in primate retina was concentrated not only at the CC/TZ but also in cone outer segments and was weakly expressed at rod outer segments and cone terminals.** Eye cups from three 8-year-old female rhesus macaque monkey and a human donor of undisclosed age and sex were stained with antibodies against EYS, acetylated α-tubulin, rhodopsin, or blue opsin and PNA. (A-C) Staining with no primary antibody, EYS antibody, and EYS antibody and blocking peptide, respectively. Note EYS immunoreactivity was observed as punctate pattern (arrow), cone outer segment (arrowhead), and rod outer segment (# signs) between the RPE and ONL as well as cone terminals (asterisk). (D,E) Double immunostaining of EYS (green) and acetylated α-tubulin (red). Note EYS reactivity was located on the basal end of acetylated tubulin (arrows), rod outer segment (asterisks), and adjacent to cone axonemes (arrowheads). (F) Double staining of EYS antibody (green) with rhodopsin antibody 1D4 (red). EYS is concentrated in the position basal to 1D4 immunoreactivity (arrow) and some of the EYS reactivity overlapped with 1D4. (G) Double staining of EYS antibody (green) with PNA (red). Note that EYS immunoreactivity in the cone outer segment was completely wrapped by PNA-stained cone matrix. EYS immunoreactivity in the OPL was also reactive to PNA (arrowheads). Insert in G shows a single cone at higher magnification. (H-J) Double staining of EYS (green) and blue opsin (red) antibodies. Note that a subset of EYS-positive cone outer segment was immunoreactive to blue opsin antibody (arrows) and there is a complete overlap between EYS and blue opsin reactivities (I,J). (K-M) Double staining of EYS (green) and acetylated α-tubulin (red) of human retina. Note that EYS immunoreactivity was located on the basal end of acetylated α-tubulin immunoreactivity (arrows). (N) Some human EYS immunoreactivity was located within PNA-positive domain. Scale bar in C: 20 µm for A-C; scale bar in J: 2 μm for E,F,I,J,L-N; 5 μm for D; 10 μm for K; 20 μm for G,H. IS, inner segment; INL, inner nuclear layer; ONL, outer nuclear layer; OS, outer segment; PNA, peanut agglutinin; RPE, retinal pigment epithelium.

Our results in the zebrafish retina differ from those in the porcine retina where EYS was shown to be co-localized with rhodopsin (Abd El-Aziz et al., 2008). To determine the distribution of EYS in primate retinas, we carried out immunofluorescence staining of rhesus macaque monkey and human retinal sections. In the monkey retina, punctate immunoreactivity similar to that of the zebrafish was observed between the ONL and the RPE (Fig. 2B, arrow); however, strong immunoreactivity was also observed in what appeared to be the cone outer segment (Fig. 2B, arrowhead). Weak immunoreactivity was also observed at the cone terminal (Fig. 2B, asterisk) and rod outer segment (Fig. 2B, indicated by #). These immunoreactive patterns were not observed in controls lacking the EYS antibody (Fig. 2A) or when the EYS antibody was blocked with the peptide used to generate the antibody (Fig. 2C). Double immunostaining of EYS and acetylated α-tubulin antibodies revealed that the punctate immunoreactivity was located on the basal end of acetylated α-tubulin signals (Fig. 2D,E, arrows). Weak immunoreactivity was also observed on the sides of acetylated α-tubulin (Fig. 2D,E, asterisks), which was completely covered by immunoreactivity to the 1D4 antibody (Fig. 2F), indicating that this immunoreactivity was located in the rod outer segments. EYS in the cone outer segment was opposed to acetylated α-tubulin (Fig. 2D, arrowheads) and was within the PNA-stained cone inter-photoreceptor extracellular matrix sheath (Fig. 2G). Some EYS immunoreactive staining patterns completely overlapped with those of anti-blue opsin immunoreactive staining (Fig. 2H-J, arrows), indicating that the EYS protein was highly concentrated in the cone outer segment. EYS immunoreactivity within the outer plexiform layer (Fig. 2G, arrowheads) was also reactive to PNA, indicating that EYS was weakly expressed at cone terminals. These results indicate that EYS in the monkey retina is not only highly concentrated near the CC/TZ, as in zebrafish, but also highly concentrated in cone outer segments. Additionally, it is also weakly expressed in the rod outer segment and the cone terminals in primate but not zebrafish retina.

To evaluate whether EYS in the human retina is localized in a pattern similar to the monkey retina, we double stained human retina using the EYS antibody with either acetylated α-tubulin or PNA. Although the human retinal sample was not preserved as well as the monkey retina, EYS punctate immunoreactivity was observed on the basal end of acetylated α-tubulin (Fig. 2K,L) and within PNA-stained cones (Fig. 2N) between the RPE and outer nuclear layer. These results indicate that EYS in the human retina is expressed near the CC/TZ as well as in the cone outer segment.

### Disruption of the cone photoreceptor ciliary pocket in mutant EYS zebrafish

To study the roles of EYS in photoreceptor health, we generated mutations in zebrafish EYS by gene editing with CRISPR/Cas9 (see Materials and Methods section for details; Table S1). To reduce the likelihood that the observed phenotypes were derived from off-target CRISPR effects, we evaluated multiple mutant lines and backcrossed the mutant lines with the wild-type animals. Homozygous mutant animals of both *eys^sny10^* and *eys^sny14^* mutant lines exhibited identical phenotypes when backcrossed two generations to the wild-type animals. In addition, homozygous mutant animals of *eys^sny13^* exhibited identical phenotypes as *eys^sny10^* and *eys^sny14^*. All experiments in this report were carried out using two mutant alleles, *eys^sny10^* and *eys^sny14^*. Heterozygous animals analyzed at all ages did not show any differences from the wild type.

**Fig. 3.**
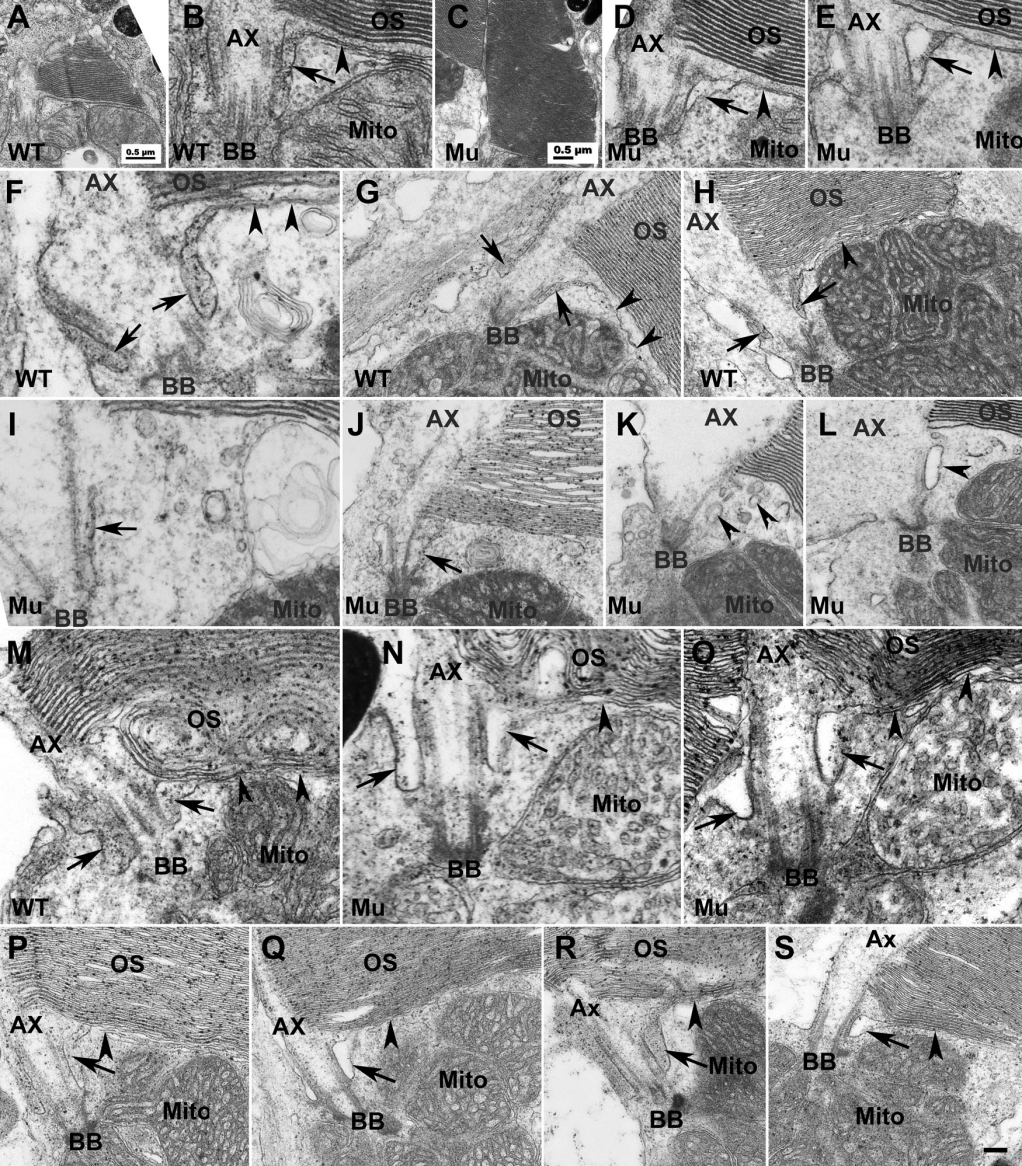
**EYS deficiency caused disruption of the ciliary pocket in cones.** To determine the impact of EYS deficiency on structures near CC/TZ, we performed EM analysis on EYS-deficient zebrafish retina at 7, 40, 50, 80 dpf and 8 and 14 mpf. (A,B) Low and high magnification of same wild-type cone photoreceptor at 7 dpf. (C,D) Low and high magnification of the same EYS-deficient cone photoreceptor at 7 dpf. (E) Another example of EYS-deficient cone photoreceptor at 7 dpf. (F-H) Wild-type cone photoreceptors at 40 dpf. (I-L) EYS-deficient zebrafish cone photoreceptors at 40 dpf. (M) Wild-type rod photoreceptor at 40 dpf. (N,O) EYS-deficient rod photoreceptors at 40 dpf. (P) Wild-type rod photoreceptor at 8 mpf. (Q) EYS-deficient rod photoreceptor at 8 mpf. (R) Wild-type rod photoreceptor at 14 mpf. (S) EYS-deficient rod photoreceptor at 14 mpf. In wild type at 7 and 40 dpf cones, the ciliary pocket with electron dense lumen was clearly observed (B,F-H, arrows). The plasma membrane separating the outer and inner segments was in continuity with the membrane of the ciliary pocket (arrowheads in B,F-H). In EYS-deficient animals at 7 dpf, the cone ciliary pocket was normal except that the lumen of the mutant ciliary pocket was not as electron dense as the wild type (D,E, arrows). At 40 dpf, in EYS-deficient animals, the ciliary pocket in cone photoreceptors was collapsed (I and J, arrows) or was replaced with multiple membrane vesicles of electron density (K,L, arrowheads). The rod ciliary pocket in EYS-deficient zebrafish was largely maintained (M-O,Q,S, arrows). Scale bar in S: 100 nm for B,D-F,I,M-O; 200 nm for G,H,J,L,P-S. Ax, axoneme; BB, basal body; Mito, mitochondria; OS, outer segment.

To determine the impact of EYS deficiency on structures near the CC/TZ, we performed EM analysis on zebrafish retinas at several different ages, including 7, 40, 50, and 80 days post-fertilization (dpf) as well as 8 and 14 months post-fertilization (mpf) with special emphasis on the inner and outer segment junction. At 7 dpf most of the mature photoreceptors were cones, therefore we focused on analysis of cone photoreceptors at this age. In the wild-type animals (Fig. 3A,B, low and high magnification of the same cone), a ciliary pocket was clearly identified with an electron dense extracellular lumen near the CC/TZ (Fig. 3B, arrow). All 18 cone photoreceptors imaged from three wild-type animals exhibited similar ciliary pocket morphology. The ciliary pocket plasma membrane was in continuity with the plasma membrane that separated the outer and the inner segments (Fig. 3B, arrowhead). In EYS-deficient animals (Fig. 3C,D, low and high magnification of the same cone; Fig. 3E, another cone) a ciliary pocket was also clearly identified but the lumen appeared less electron dense. All 15 cone photoreceptors from three EYS-deficient animals exhibited similar ciliary pocket morphology.

At 40 dpf, in wild-type animals, a ciliary pocket with an electron dense extracellular lumen was clearly identified as in 7 dpf zebrafish near the CC/TZ in cone photoreceptors (Fig. 3F-H, arrows). The ciliary pocket plasma membrane was continuous with the plasma membrane that separated the outer and the inner segments (Fig. 3F-H, arrowheads). In EYS-deficient animals, the ciliary pocket of cone photoreceptors was either collapsed (Fig. 3I-J, arrows) or replaced with membrane vesicles (Fig. 3K-L, arrowheads). The lumen of these vesicles was not as electron dense as the lumen of the ciliary pocket in the wild-type animals. The plasma membrane in continuity with the ciliary pocket, which separates the outer and inner segments, was not observed. To quantify this effect, we obtained EM images at the CC/TZ from multiple photoreceptors in three wild-type and three EYS-deficient zebrafish. In 32 wild-type cones, 31 showed ciliary pockets with electron dense lumen. The plasma membrane in continuity with the ciliary pocket, separating the inner and outer segment, was identified. Only one cone did not show a clearly discernable ciliary pocket but showed multiple vesicles. In 26 EYS-deficient retinas, eight showed collapsed ciliary pockets, 13 showed multiple vesicles near the position of ciliary pocket, and five showed normal ciliary pocket morphology with lower electron density (*P*=1.36×10^−9^, Chi-square analysis). Similar results were observed for 50 and 80 dpf zebrafish.

We also evaluated the structure near the CC/TZ of rod photoreceptors in 40, 50, and 80 dpf and 8 and 14 mpf zebrafish. Similar results were obtained for all ages. At 40 dpf in the wild type, ciliary pockets with an electron dense lumen (Fig. 3M, arrows) and the plasma membrane (Fig. 3M, arrowheads) separating the outer and inner segments were clearly identified in rod photoreceptors. Similar results were obtained for 8 mpf (Fig. 3P) and 14 mpf (Fig. 3R) wild-type zebrafish. In EYS-deficient animals, the ciliary pockets were as easily recognizable as in the wild-type animals, although their lumen was not as electron dense (Fig. 3N,O,Q,S). At 40 dpf, 12 of 16 wild-type rods (from three animals) showed clear ciliary pocket morphology and 12 of 14 EYS-deficient rods (from three animals) showed a ciliary pocket with morphology that was indistinguishable from wild type (*P*=0.464, Chi-square analysis). At 14 mpf, 22 of 25 wild-type rods (from three animals) showed normal ciliary pocket morphology and 16 of 20 EYS-deficient rods (from four animals) showed a ciliary pocket with morphology that was indistinguishable from the wild type (*P*=0.6822, Chi-square analysis). These results indicate that EYS is required for maintaining the normal structure of the ciliary pocket in cones.

### Cone photoreceptors degenerated in EYS-deficient zebrafish at 6 to 8 mpf

**Fig. 4.**
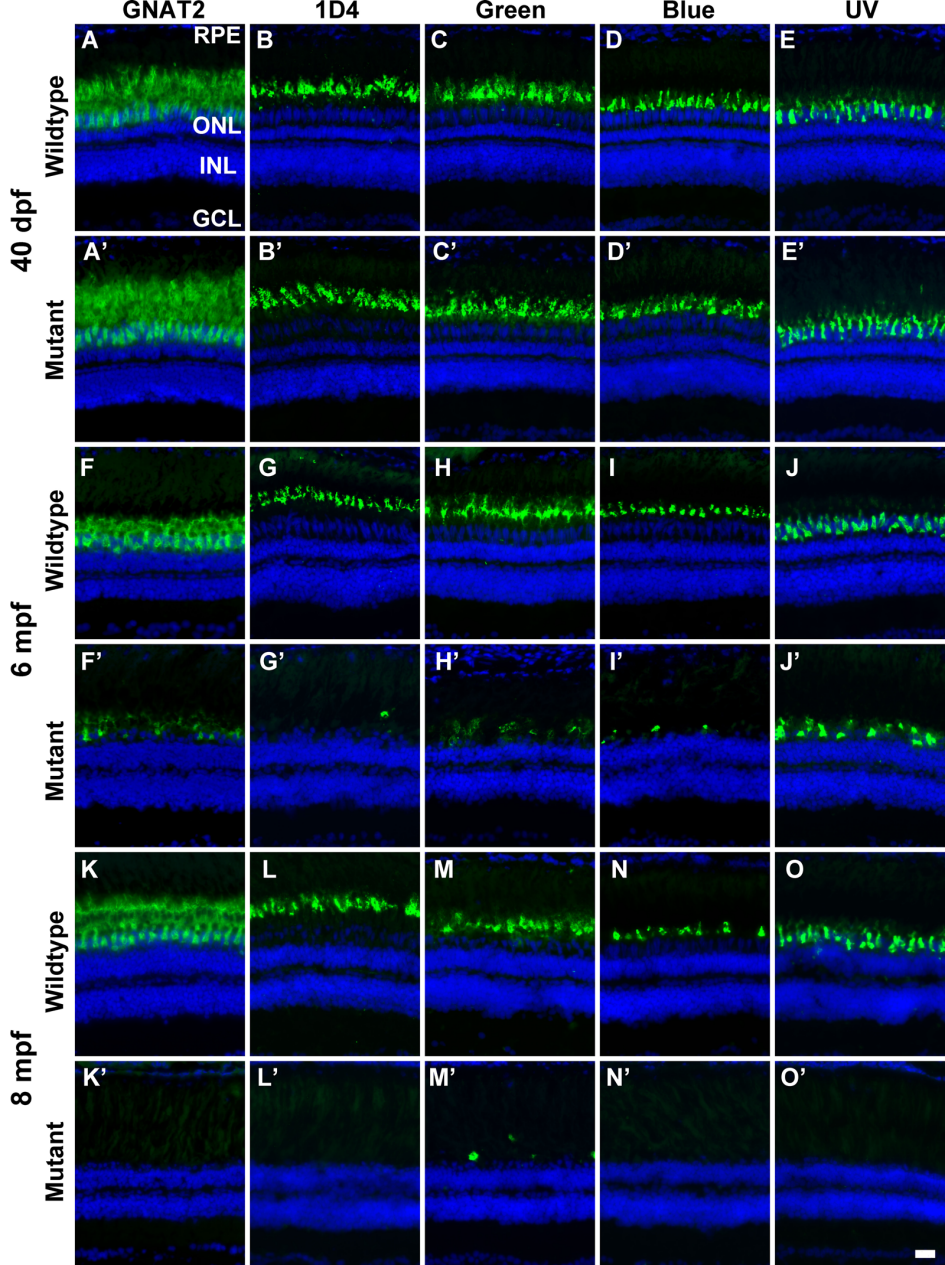
**EYS-deficient zebrafish exhibited degeneration of cone photoreceptors.** Zebrafish retinal sections were immunostained with GNAT2, 1D4, green opsin, blue opsin, and UV opsin antibodies. (A-E′) GNAT2, 1D4, green opsin, blue opsin, and UV opsin staining of 40 dpf wild-type and EYS-deficient zebrafish. There were no apparent differences between wild-type and EYS-deficient zebrafish. (F-J′) GNAT2, 1D4, green opsin, blue opsin, and UV opsin staining of 6 mpf wild-type and EYS-deficient zebrafish. Note GNAT2-, 1D4-, green opsin-, blue opsin-, and UV opsin-positive immunoreactivity was reduced in EYS-deficient zebrafish. (K-O′) GNAT2, 1D4, green opsin, blue opsin, and UV opsin staining of 8 mpf wild-type and EYS-deficient zebrafish. Note that GNAT2-, 1D4-, green opsin-, blue opsin-, and UV opsin-positive immunoreactivity was further reduced in EYS-deficient zebrafish. Scale bar: 20 µm.

We first evaluated the impact of EYS deficiency on zebrafish retina at 40 dpf and 2, 6, 8.5 and 14 mpf by H&E staining (Fig. S3). While no apparent difference was observed at 40 dpf and 2 mpf, progressive loss of cone photoreceptors was apparent in 6, 8.5 and 14 mpf EYS-deficient zebrafish. To further evaluate the impact of EYS deficiency on cone photoreceptors, we carried out immunostaining of wild-type and knockout retinal sections with a guanine nucleotide binding protein (G protein) α transducin activity polypeptide 2 (GNAT2) antibody. At 40 dpf, GNAT2 immunoreactivity in the mutant and wild-type retinas was indistinguishable (Fig. 4A,A′). No difference in GNAT2 immunoreactivity was observed at 2 mpf either (data not shown). By 6 and 8 mpf, however, GNAT2 immunoreactivity was significantly and progressively diminished in the mutant retina (Fig. 4F,F′,K,K′), indicating the loss of cone photoreceptors. To identify the type of cone photoreceptors lost, we immunolabeled zebrafish retinas with green-, blue-, and UV-cone-specific antibodies, as well as a 1D4 antibody. Although 1D4 recognizes the rod outer segment in other species, it labels red cones in zebrafish (Yin et al., 2012). For markers of the four cone types, no significant difference was observed between 40 dpf mutant and wild-type zebrafish (Fig. 4B-E′). In contrast, immunoreactivity to all four cone markers was significantly reduced in 6 and 8 mpf mutant retinas when compared to respective wild-type retinas (Fig. 4G-J′; Fig. 3L-O′). This indicates that all four types of cone photoreceptors were progressively lost in EYS-deficient zebrafish.

**Fig. 5.**
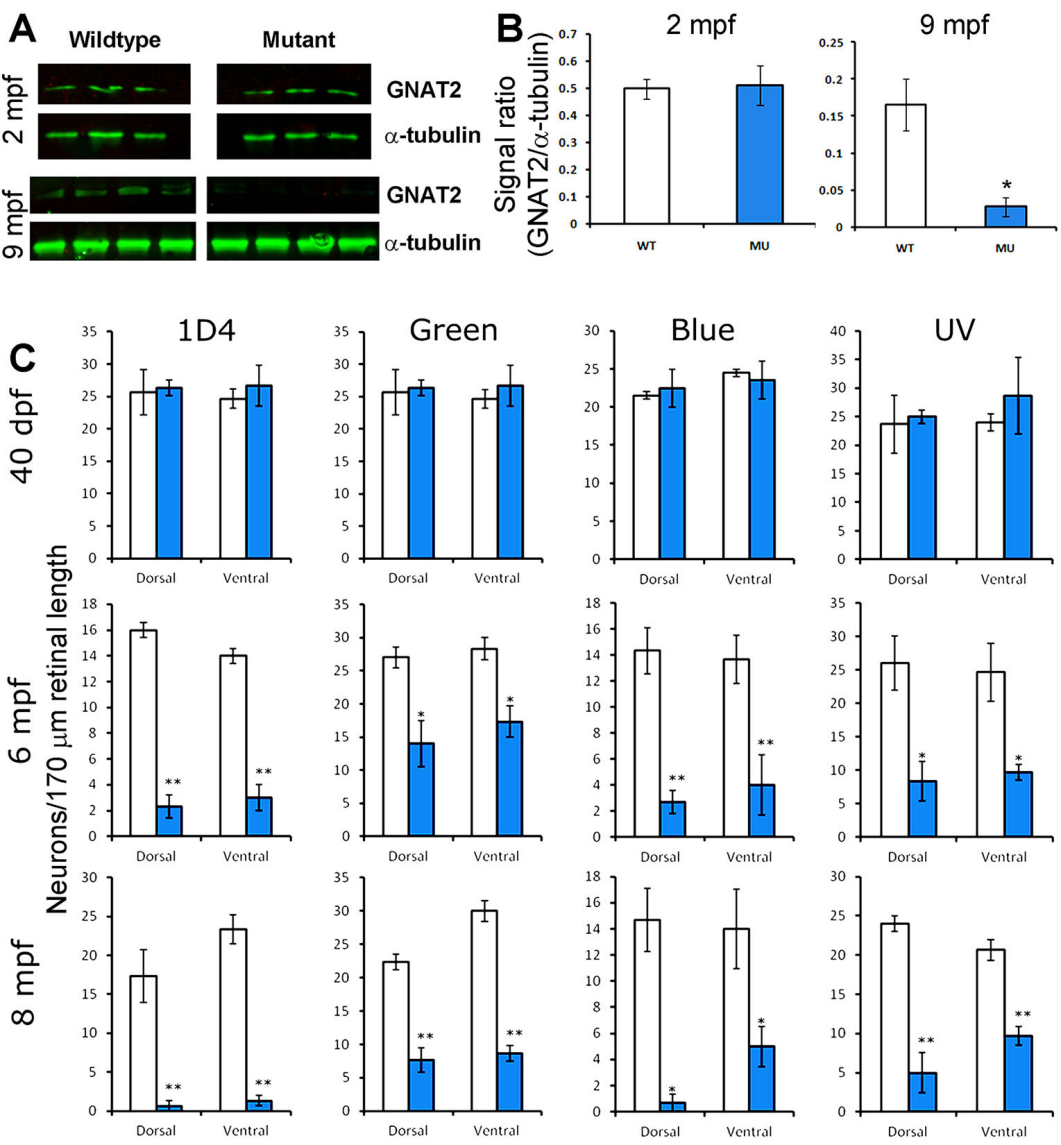
**Reduction of GNAT2 protein and cone density in EYS-deficient zebrafish.** Western blotting for GNAT2 was performed on retinal lysates from 2 mpf and 9 mpf zebrafish. Cone density was determined by counting 1D4-, green opsin-, blue opsin-, and UV opsin-positive cells in the dorsal and ventral retina. (A) Western blot for GNAT2 and α-tubulin. Note that while GNAT2 signal was not changed in 2 mpf mutant zebrafish, it was diminished in 9 mpf mutant zebrafish. (B) Quantification of western blot shown in A. *P<0.05, Student's *t*-test, two-tailed. Error bars indicate SEM (standard error of the mean). (C) Cone density in EYS-deficient zebrafish was normal at 40 dpf but reduced at 6 and 8 mpf. *P<0.05; **P<0.01 (repeated measures ANOVA). Error bars indicate s.e.m.

To quantify the loss of cones, we performed GNAT2 western blots on retinal lysates from 2 and 9 mpf mutant zebrafish (Fig. 5A,B). The EYS-deficient retinas did not have a different GNAT2 protein level than the wild type at 2 mpf. However, GNAT2 protein level in EYS-deficient retinas was significantly reduced compared to that in the wild type at 9 mpf and became undetectable at 14 mpf. We also counted the density of red, green, blue, and UV cones at identical locations in the dorsal and ventral retinas of wild-type and mutant zebrafish (Fig. 5C). The density of all four types of cones in EYS-deficient zebrafish was not significantly different from the wild type at 40 dpf. In 6 and 8 mpf EYS-deficient zebrafish, however, the density of all four types of cones was significantly reduced compared to the wild type (*n*=3 for each group). We also performed real-time RT-PCR for red opsin, green opsin, blue opsin, and UV opsin mRNAs with total RNA extracts from wild-type and mutant zebrafish retinas using β-actin mRNA as an internal control. While no significant changes were observed for 40 dpf mutant retinas, all of the four opsin mRNAs were significantly reduced compared to the wild type in 8 mpf EYS-deficient zebrafish (data not shown).

### Rod photoreceptors degenerated in older but not in younger EYS-deficient zebrafish

**Fig. 6.**
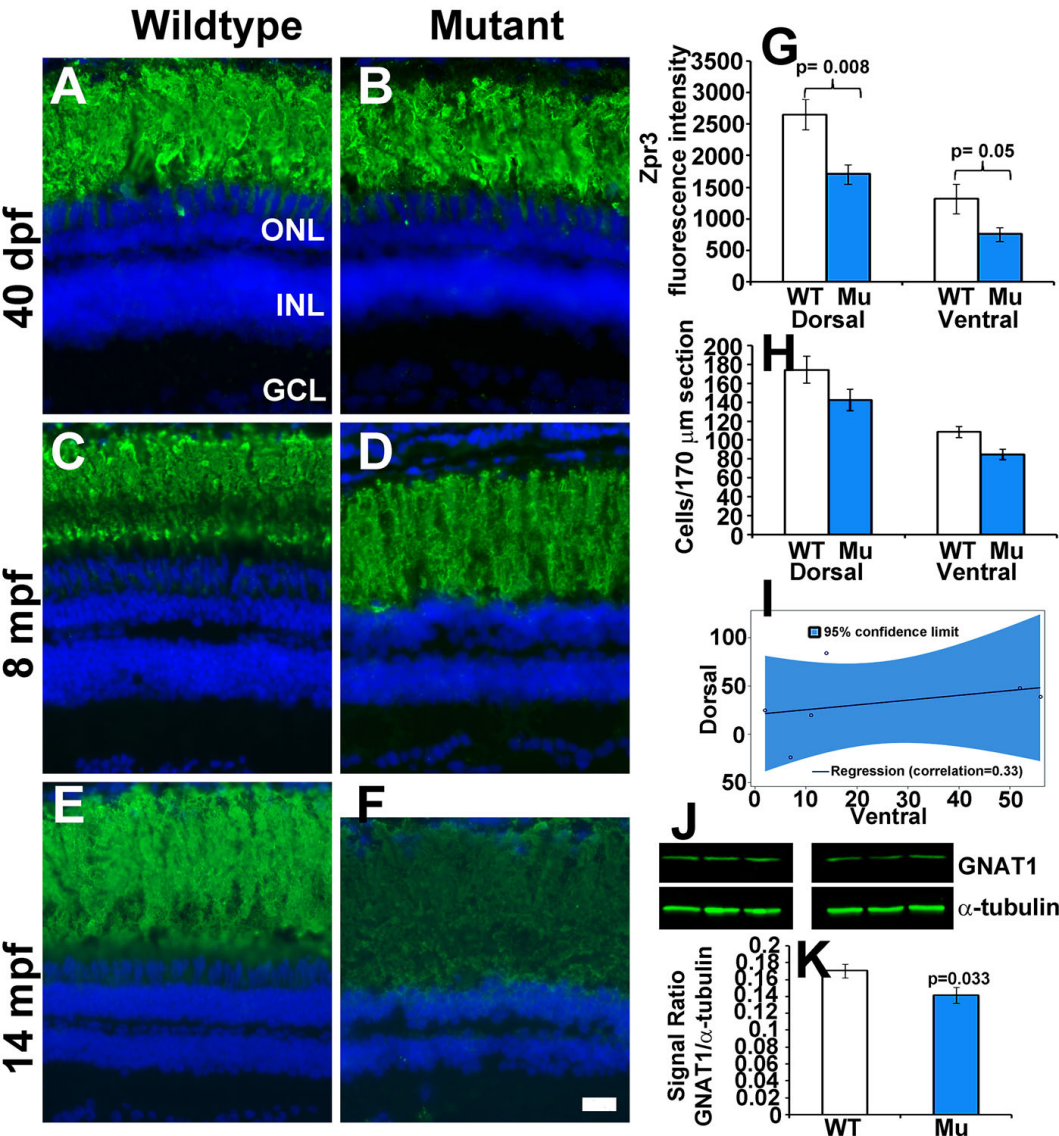
**Rod photoreceptors degenerate in older EYS-deficient zebrafish.** (A-F) Retinal sections were stained with Zpr3 and counter stained with DAPI. (A,B) Zpr3 staining of wild-type and EYS-deficient zebrafish at 40 dpf. (C,D) Zpr3 staining of wild-type and EYS-deficient zebrafish at 8 mpf. (E,F) Zpr3 staining of wild-type and EYS-deficient zebrafish at 14 mpf. (G) Quantification of Zpr3 immunofluorescence intensity of wild-type and EYS-deficient zebrafish at 14 mpf. (H,I) Rod quantification in wild-type and EYS-deficient zebrafish at 14 mpf. Rod cells in dorsal and ventral retina near the optic nerve heads were counted on H&E stained sections from sex- and body weight-matched animals. (J,K) GNAT1 western blot and quantification at 14 mpf. Western blot was carried out with GNAT1 and α-tubulin antibodies. Note that no significant differences were observed between wild-type and EYS-deficient zebrafish at 40 dpf and 8 mpf. However, Zpr3 immunoreactivity, rod cell numbers and GNAT1 protein were reduced in EYS-deficient zebrafish at 14 mpf. Scale bar for A-F: 20 µm. Error bars in G,H,K indicate s.e.m.

To evaluate whether rod photoreceptors were impacted by the loss of EYS in zebrafish, we carried out immunostaining with the Zpr3 antibody, a rod marker (Yokoi et al., 2009). No significant difference was observed between 40 dpf and 8 mpf EYS-deficient and wild-type zebrafish (Fig. 6A-D). However, Zpr3 immunoreactivity was reduced in 14 mpf EYS-deficient zebrafish (*n*=6, sex and body-weight matched pairs, Fig. 6E,G). We also counted the rod nuclei in dorsal and ventral retina near the optic nerve head. While no significant difference was observed in 2, 6 and 8.5 mpf EYS-deficient zebrafish (Fig. S4), rods were reduced in 14 mpf EYS-deficient zebrafish (Fig. 6H,I, *n*=6, sex and body-weight matched pairs, *P*=0.0054, Wald-type test for the average of the correlated rod reduction in dorsal and ventral retinas). As expected, rod reduction in dorsal and ventral retinas was highly correlated (Fig. 6I, correlation coefficient=0.33). We also carried out GNAT1 western blots on retinal lysates from 2, 9 and 14 mpf wild-type and mutant zebrafish. While there was no significant difference in GNAT1 protein levels between wild-type and mutant samples at 2 and 9 mpf, GNAT1 protein was moderately reduced in 14 mpf EYS-deficient zebrafish (Fig. 6J,K, *P*=0.033, *n*=9, Student's *t*-test, two-tailed). These results indicate that rod photoreceptors degenerate in older EYS-deficient zebrafish.

### TUNEL-positive cells were observed in EYS-deficient retina

**Fig. 7.**
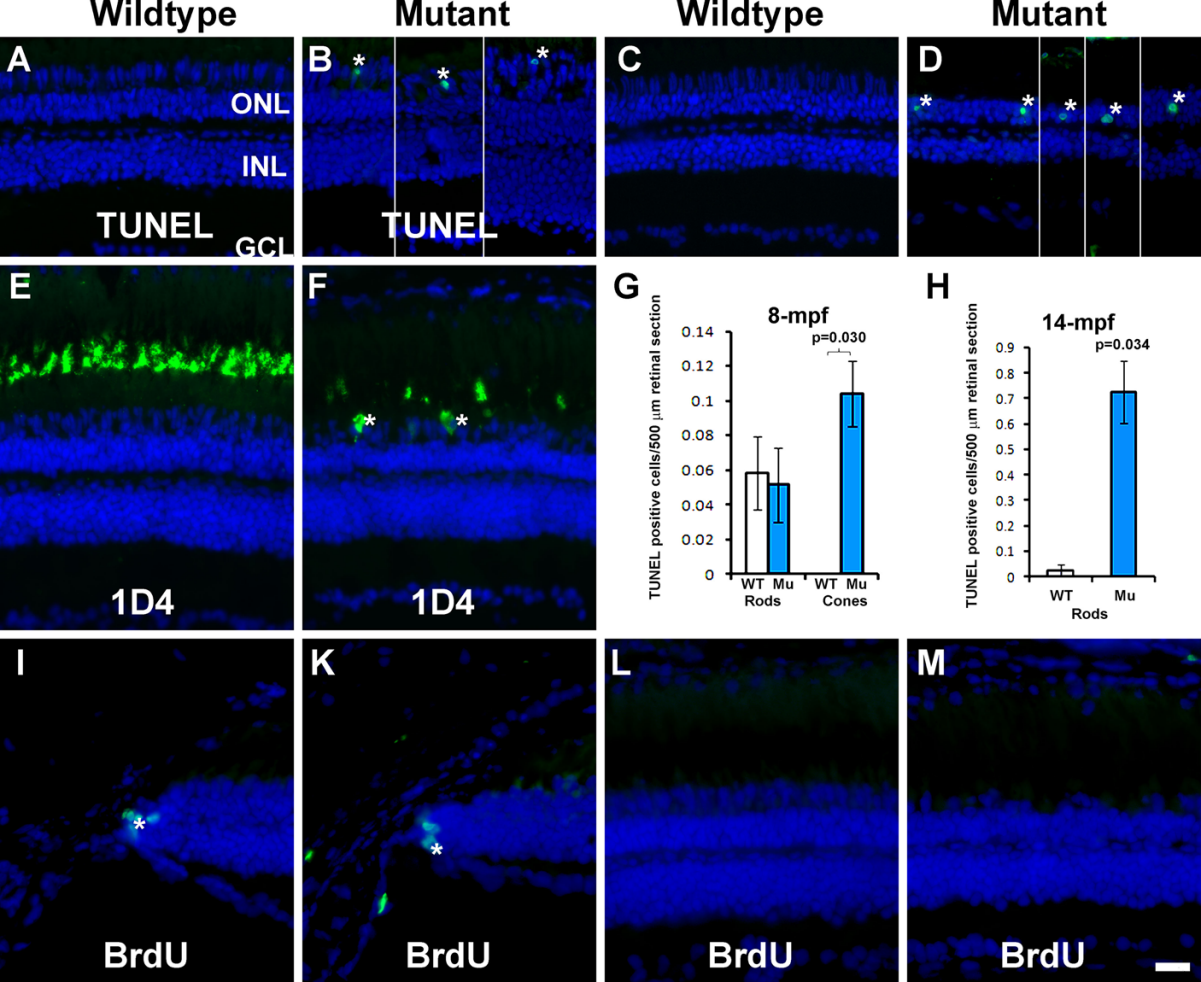
**TUNEL-positive cells were observed in some cone and rod nuclei and BrdU incorporation were not changed in EYS-deficient retina.** TUNEL staining and 1D4 immunostaining were carried out on retinal sections of 4, 8, and 14 mpf wild-type and EYS-deficient zebrafish retinas. BrdU-labeled nuclei were detected by immunofluorescence staining. (A) TUNEL staining of wild-type retina at 4 mpf. (B) TUNEL staining of EYS-deficient retina at 4 mpf. (C) TUNEL staining of wild-type retina at 14 mpf. (D) TUNEL staining of EYS-deficient retina at 14 mpf. (E) 1D4 immunostaining of wild-type retina. (F) 1D4 immunostaining of EYS-deficient retina. (G,H) Quantification of TUNEL-positive nuclei at 8 and 14 mpf, respectively. Error bars indicate s.e.m. (I,K) BrdU labeling at the ciliary margin of wild-type and EYS-deficient retina, respectively. (L,M) BrdU labeling at the central retina of wild-type and EYS-deficient retina, respectively. Note the presence of TUNEL-positive cone nuclei (asterisks in B), TUNEL-positive rod nuclei (asterisks in D), and 1D4 immunoreactive long double cone soma (asterisks in F) in EYS-deficient retinas. Also note absence of BrdU-labeled nuclei in the central retina of EYS-deficient animals. Scale bar: 20 µm.

To determine whether photoreceptors in EYS-deficient zebrafish die via apoptosis, we carried out TUNEL staining. TUNEL-positive cone nuclei were observed in EYS-deficient zebrafish (Fig. 7B, asterisks) at 4 and 8 mpf. TUNEL-positive rod nuclei were also observed in EYS-deficient zebrafish at 14 mpf (Fig. 7D, asterisks). Dying photoreceptors often exhibit mis-localization of outer segment proteins at the cell soma. Indeed, 1D4-immunoreactivity was exclusively located in the outer segment in wild-type retina (Fig. 7E); however, some 1D4-immunoreactivity was observed at the cell soma of the outer nuclear layer in 4 mpf EYS-deficient zebrafish (Fig. 7F, asterisks). We counted TUNEL-positive cone and rod nuclei at 8 mpf (Fig. 7G, *n*=3). We also counted TUNEL-positive rod nuclei at 14 mpf because cones were absent in the central retina at this age (Fig. 7H, *n*=3). TUNEL-positive nuclei were significantly increased in cones at 8 mpf (*P*=0.03, Student's *t*-test, two-tailed) and in the rods at 14 mpf (*P*=0.034, Student's *t*-test, two-tailed) in EYS-deficient zebrafish.

We also evaluated whether there is an increase in cell proliferation to regenerate the lost cones by BrdU incorporation assay. Although BrdU-labeled nuclei were found consistently at the ciliary margin of the wild-type and EYS-deficient retinas at 8.5 mpf (Fig. 6E,F, asterisks), they were rarely found in the central retina near the optic nerved heads of either genotype (Fig. 6G,H). We counted BrdU-labeled nuclei in the ciliary margin and the central retina. There was no significant difference between the EYS-deficient and wild-type retinas in the ciliary margin as well as in the central retina (Table 1). Together, these results indicate that, in EYS-deficient zebrafish, all four types of cones as well as rods degenerate, with rods degenerating after the loss of cones**.**

**Table 1.**
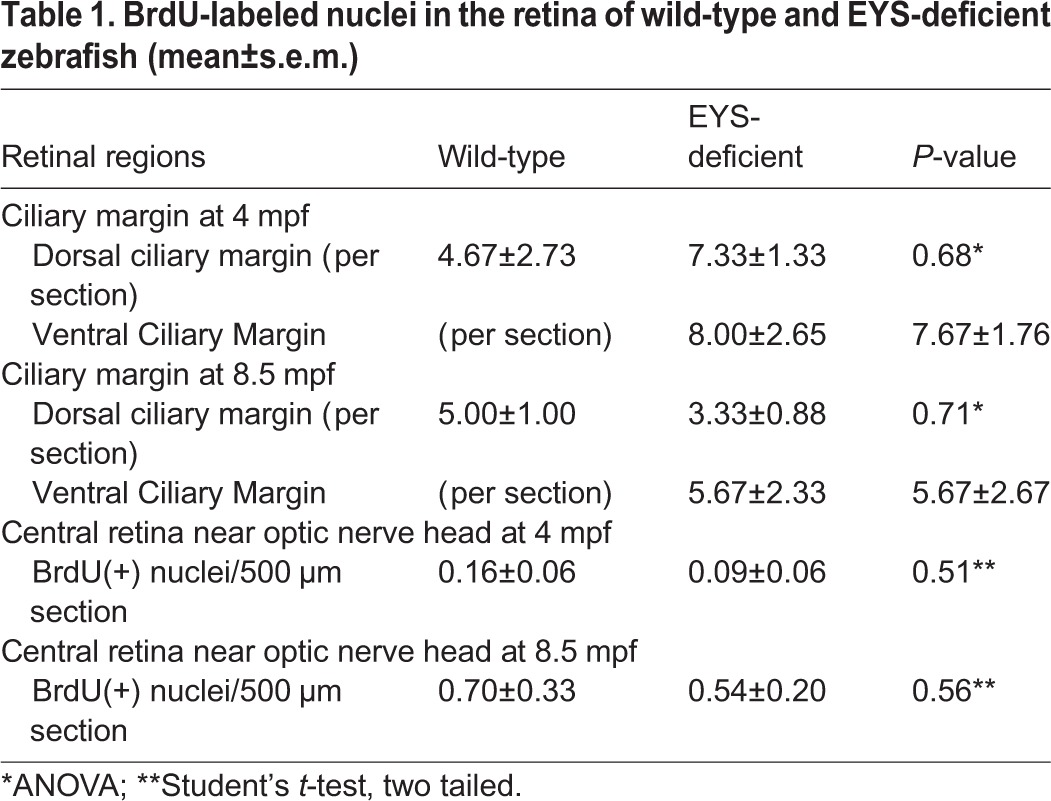
**BrdU-labeled nuclei in the retina of wild-type and EYS-deficient zebrafish (mean±s.e.m.)**

## DISCUSSION

Mutations in EYS are known to cause photoreceptor degeneration in patients with RP25, but functions of the EYS protein in the vertebrate retina are poorly understood. Our study shows that the EYS protein localized near the CC/TZ of both rods and cones in the zebrafish retina. In primate retinas, EYS protein was not only highly concentrated near the CC/TZ, but was also located in the outer segments of cone photoreceptors and weakly expressed at the rod outer segments and cone terminals. Analyses of multiple EYS mutant zebrafish strains revealed a cone-rod pattern of photoreceptor degeneration. EM analysis on animals before the onset of degeneration indicated that EYS is required for maintaining the normal shape of the cone ciliary pocket. These results indicate EYS mutant zebrafish provide a useful system for further defining the cellular and molecular functions of the vertebrate EYS protein.

In one of the two landmark papers that identify EYS mutations as the cause of RP25 (Abd El-Aziz et al., 2008; Collin et al., 2008), Abd El-Aziz and colleagues reported that porcine EYS protein is located at the outer segment based on immunofluorescence staining with rhodopsin. Whether porcine EYS is also located near the CC/TZ is not known. In zebrafish, our results indicated that EYS was located near the CC/TZ. In the primate retina, EYS was not only located near the CC/TZ but also in the cone outer segments, rod outer segments, and cone terminals. Could the EYS protein also be located in the zebrafish outer segment? While this remains a possibility, the immunofluorescence intensity of the EYS antibody in the outer segment of wild-type animals was similar to the background fluorescence of homozygous mutant animals. This indicates that zebrafish EYS is not present at high levels in the outer segment. Our results suggest that the EYS protein in vertebrate retinas has evolved different functions since it exhibits species-specific distribution patterns. Besides being critical for ciliary pocket morphology, the differential distribution patterns of EYS protein in different vertebrates suggests a high likelihood that the protein may have evolved to have different or multiple roles in different species to maintain photoreceptor health.

Although EYS is required for the development of the inter-rhabdomeral space in the Drosophila eye and photoreceptor survival in the human retina, several mammals, including mouse, lack an EYS gene (Abd El-Aziz et al., 2008). Furthermore, an EYS locus is not present in the *Xenopus laevis* genome. Consistent with this, we found that retinal sections from *X. laevis* did not show EYS immunoreactivity (data not shown). It is interesting to consider how photoreceptors in the vertebrate species lacking EYS maintain survival. We speculate that other molecule(s) compensate for the absence of EYS in these species. Future studies should be aimed at identifying such molecules, since the identification of compensatory factors may have therapeutic implications for RP25 patients lacking EYS.

**Fig. 8.**
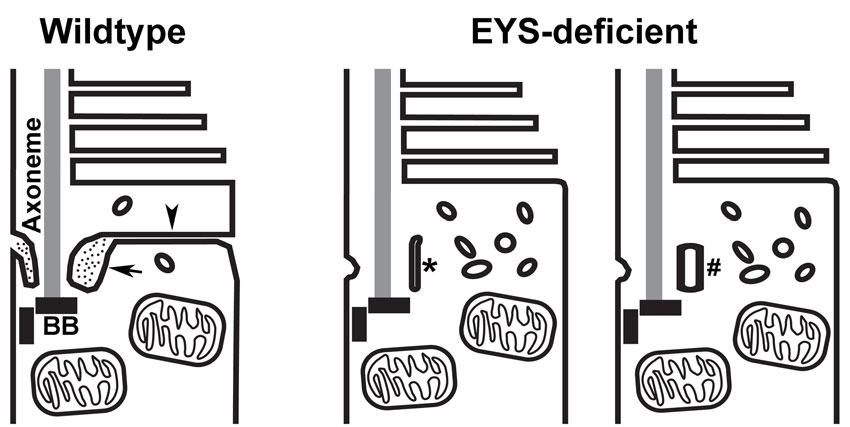
**Model of EYS function at the CC/TZ.** In the wild-type photoreceptors, the lumen of the ciliary pocket is electron dense (arrow). The ciliary membrane and the plasma membrane of the ciliary pocket are in continuity with the plasma membrane that demarcates the inner and the outer segments (arrowhead). In EYS-deficient cone photoreceptors, the ciliary pocket is either collapsed (asterisk) or replaced with vesicles (#). The membrane in continuity with the ciliary pocket that demarcates the inner and outer segments is not observed.

About 25% of the offending genes in photoreceptor degeneration are associated with the structure and function of cilia (Wright et al., 2010). Based on confocal and immuno-electron microscopic studies largely in the mouse retina, retinitis pigmentosa-associated ciliary proteins are concentrated at the axoneme, CC/TZ, periciliary complex/ciliary pocket, or basal bodies (reviewed in Rachel et al., 2012). In mouse photoreceptors, several protein complexes involved in Usher syndromes, a group of genetic diseases that exhibit photoreceptor degeneration, are localized at or near the CC/TZ including Usher 1 syndrome (USH1) proteins (Reiners et al., 2006), USH2 proteins (Chen et al., 2014; Ebermann et al., 2007; Eudy et al., 1998; Liu et al., 2007; Mburu et al., 2003; Wang et al., 2012; Weston et al., 2004), and the USH3 protein clarin-1 (Zallocchi et al., 2009). In addition, Meckel–Gruber syndrome (MKS) proteins (Huang et al., 2011; Williams et al., 2011), nephronophthisis (NPHP) proteins (Sang et al., 2011), and tectonic complexes (Garcia-Gonzalo et al., 2011) are located at the transition zone. Interestingly, there are species variations regarding localization of Usher syndrome proteins. In contrast to the mouse, primate USH1 proteins are located in the calycal processes that are absent in mouse photoreceptors (Sahly et al., 2012) and USH1B (Myosin VIIa) is located in the accessory outer segment in zebrafish (Hodel et al., 2014). Nevertheless, all of the members in these complexes at or near the CC/TZ are either intracellular or transmembrane proteins. Our data indicate that the extracellular matrix protein EYS is concentrated near the CC/TZ of zebrafish and primate photoreceptors. We hypothesize that EYS near the CC/TZ is localized in the lumen of the ciliary pocket. This hypothesis is supported by the finding that the ciliary pockets of cone photoreceptors are disrupted in EYS-deficient zebrafish and by the fact that EYS is a secreted extracellular matrix protein. Thus, our results are consistent with a model that EYS is localized in the lumen of the ciliary pocket of photoreceptors where it functions to maintain the normal structure of the ciliary pocket and the plasma membrane continuity of the ciliary pocket that demarcates the outer and inner segments of cone photoreceptors (Fig. 8). It will be essential to determine the precise location of EYS protein with respect to the CC/TZ in order to further explore EYS function. We attempted immuno-EM studies with the EYS antibody, but despite using various pre- and post-embedding procedures the EYS antibody did not provide specific signals. Generation of new EYS antibodies and transgenic zebrafish expressing EYS-EGFP fusion protein will be needed to determine the precise localization of EYS protein by immuno-EM.

Disruption of the cone ciliary pocket was found to precede cone photoreceptor degeneration in EYS-deficient zebrafish. Though we did not find an increase in the disruption of rod ciliary pockets, we cannot rule out a structural impact on the rod ciliary pocket because there is a larger number of rods than cones in zebrafish and because rods degenerate at much slower rate than the cones in EYS-deficient zebrafish. It remains possible that rod degeneration in EYS-deficient zebrafish is caused by a disruption of the rod ciliary pocket, or alternatively, by the loss of cones that deprives the rods of a survival factor(s).

In *Drosophila*, EYS deficiency results in the closing or collapse of the inter-rhabdomeral space (Husain et al., 2006; Zelhof et al., 2006). Our observations of collapsed ciliary pockets in EYS-deficient zebrafish cones suggest that EYS plays an evolutionary conserved structural role as an extracellular matrix molecule. Our results also suggest an important role for the extracellular matrix near the CC/TZ in maintaining ciliary pocket morphology and photoreceptor health. It remains unclear what proteins interact with EYS in the extracellular matrix. Genetic interaction studies in *Drosophila* suggest EYS interacts with the transmembrane protein prominin 1 (Zelhof et al., 2006). We speculate that EYS may physically interact with prominin-1 and/or with protein complexes known to localize near the CC/TZ. The presence of multiple EGF and laminin G domains also strongly supports the existence of binding partners. Future studies aimed at identifying EYS interacting proteins will provide further insight into EYS functions.

As a diurnal animal with cone-dominated vision, the zebrafish has become a prominent model to study photoreceptor degeneration caused by ciliopathies. For example, forward genetic screens have identified mutations in several ciliary proteins, including intraflagellar transport (IFT) proteins such as Ift88, Ift172, Ift57 and Cluap1, that impact photoreceptor survival (Gross et al., 2005; Lee et al., 2014; Tsujikawa and Malicki, 2004). Knockdown of Ift172 results in the loss of photoreceptors as well (Bujakowska et al., 2015). Reverse genetic mutations in the CC/TZ protein CC2D2A (Bachmann-Gagescu et al., 2011), axoneme protein RP2 (Liu et al., 2015), and myosin 7A (Wasfy et al., 2014) result in photoreceptor degeneration in zebrafish, indicating that zebrafish are an excellent model for the study of photoreceptor diseases associated with ciliopathies. In the case of EYS, it is difficult to model RP25 in the mouse due to the lack of an EYS gene. EYS-deficient zebrafish described here are the first animal models that exhibit photoreceptor degeneration observed in RP25 patients. Given that the zebrafish genome has been sequenced and that many pathogenic pathways are conserved between humans and zebrafish, a zebrafish model of EYS-deficiency will be a valuable tool for understanding the mechanisms underlying RP25. In addition, the zebrafish is amenable to large-scale genetic and chemical screens that can be used for therapeutic discovery.

In summary, our work provides important clues about functions of EYS in photoreceptor health and pathogenesis of photoreceptor degeneration. We show that EYS is concentrated in the extracellular matrix at the CC/TZ in vertebrate retinas and its deficiency causes the ciliary pocket to collapse in zebrafish models. To our knowledge, EYS is the first extracellular matrix protein implicated in maintaining the structural integrity of the ciliary pocket. Since components of the outer segment must be trafficked from the inner segment through CC/TZ, disruption of the ciliary pocket may therefore affect outer segment genesis or IFT functions that ultimately lead to photoreceptor death.

## MATERIALS AND METHODS

### Zebrafish maintenance

Zebrafish (AB/Tubingen strain) were obtained from Zebrafish International Resource Center, Eugene, OR, USA, and placed in recirculating water system (pH 6.6-7.4) at 26-28.5°C with a daily cycle of 14 h light:10 h dark. All experiments and handling of the animals was approved by the Institutional Animal Care and Use Committee at Upstate Medical University.

### Generation of EYS-deficient lines

The zebrafish EYS locus is a 450 kilobase region on chromosome 13. It has 47 predicted exons (Fig. S1A). Like the human EYS protein, the zebrafish EYS protein is predicted to have multiple EGF domains (39) and laminin G domains (5) according to the Simple Modular Architecture Research Tool (SMART, http://smart.embl-heidelberg.de/, Fig. S1B). Zebrafish EYS is also predicted to be a secreted protein because it encodes a signal peptide in exon 2 (amino acids 1-24, SignalP 4.1). Alignment of zebrafish and human EYS revealed that 38.25% amino acid sequences were identical between the two orthologs. The C-terminal half of the EYS molecule, which contains laminin G domains, were more highly conserved with 50.2% of residues identical between the two species.

To determine the impact of EYS deficiency on photoreceptor health, we used clustered regularly interspaced short palindromic repeats (CRISPR)/Cas9 technology to generate *eys* null mutants (Knudra Transgenics). Because the initiation codon and the signal peptide for zebrafish EYS are encoded in exon 2, three gRNAs targeting the coding region of exon 2 of the *eys* gene (Fig. S1C), GCAGAGCCACGTCTCGTGAA, TCATGGAGAACACCTGCAGC, and CCGTACACATGTCTTTGTCC, were co-injected with SpCas9 mRNA. Screening of F0 and subsequent F1 generations for mutant zebrafish was carried out by PCR with forward primer GCCCTGTGTACAGCCAGGTAAC and reverse primer CTGTAACTCCATGTTTTGCCCC, followed by gel electrophoresis to identify the presence of amplicons of smaller sizes. DNA sequencing confirmed several mutant lines containing deletions of 593, 14, 13, or 10 bp (Table S1). We have named these mutant alleles as *eys^sny593^*, *eys^sny14^*, *eys^sny13^*, and *eys^sny10^* in accordance with the convention on zebrafish mutant line designation. All of these mutations occur within exon 2 and are expected to disrupt the reading frame resulting in the loss of all EGF and laminin G domains, which is similar to the presumed pathogenic mutations L60WfsX3 (Arai et al., 2015), Q27RfsX16 (Barragán et al., 2010), T135LfsX25 (Bandah-Rozenfeld et al., 2010), and N137VfsX24 (Audo et al., 2010) found in human patients. For example, the effects on EYS proteins for *eys^sny593^*, *eys^sny14^*, and *eys^sny10^* mutations are E50SfsX11, M49QfsX47, and T47RfsX5. Thus, these frameshift mutations are presumed nulls. Table S1 lists four examples of these alleles, indicating their effects on DNA and protein sequence. RT-PCR with a pair of primers spanning the deleted nucleotides of *eys^sny14^* and *eys^sny10^* mutations showed complete absence of wild-type EYS mRNA in homozygous *eys^sny14^* and *eys^sny10^* mutant zebrafish (Fig. S2). All experiments in this report were carried out using *eys^sny10^* and *eys^sny14^* mutant lines. A total of 25 *eys^sny10^* (five at 40 dpf, three at 2 mpf, three at 80 dpf, four at 4 mpf, two at 6 mpf, five at 9 mpf, and three at 14 mpf) and 60 *eys^sny14^* (four at 30 dpf, eight at 40 dpf, eight at 2 mpf, eight at 6 mpf, 13 at 8 mpf, three at 8.5 mpf, five at 9 mpf, and 11 at 14 mpf) homozygous mutant fish and their respective wild-type or heterozygous controls were used in the experiments.

### Real-time RT-PCR

Whole eyes were isolated from zebrafish (at 1 and 9 mpf). Total RNA was extracted using RNeasy Plus Mini Kit (QIAGEN). Quantitative RT-PCR was carried out using the CFX384 Touch Real-Time PCR Detection System (Bio-Rad) with the iTaq Universal SYBR Green One-Step Kit (Bio-Rad). Primers for opn1lw1 (red opsin), opn1mw1 (green opsin), opn1sw1 (UV opsin), opn1sw2 (blue opsin), GNAT1, and actb1 (beta actin) are listed in Table S2.

### Western blot

Protein was extracted from freshly enucleated 2 mpf and 9 mpf zebrafish eyeballs with 150-300 μl of radio-immuno precipitation assay (RIPA) buffer (50 mM Tris pH 8.0, 150 mM NaCl, 1.0% NP-40, 0.5% sodium deoxycholate, 0.1% SDS) and protease inhibitor cocktail. 10 mg of total protein was separated on SDS-PAGE and transferred to PVDF membranes. After blocking with Odyssey Blocking Buffer (1:1 w/PBS; LI-COR Biosciences), the membrane was incubated with GNAT1 (GeneTex, Cat#GTX124622, 1:400 dilution), GNAT2 (1:500 dilution, MBL International, Cat#PM075), and β-tubulin (1:1000 dilution, Abcam, Cat#ab6046) antibodies in Odyssey Blocking Buffer (1:1 w/PBS+0.2%Tween 20) overnight at 4°C with gentle shaking. After washing with PBST (PBS with 0.1% Tween 20) three times, the membrane was incubated with IRDye 800cw or 680cw secondary antibodies in Odyssey Blocking Buffer (1:1 w/PBS+0.2% Tween 20+10% SDS; 1:20,000; LI-COR Biosciences) for 2 h at room temperature (RT). The membrane was then washed three times in PBST and once with PBS and was developed using an Odyssey CLx imaging system and Image Studio version 3.1 (LI-COR Biosciences). The pixel value of each band was measured using Image Studio Lite version 5.2. Quantitative analysis of the pixel value was performed using a two-tailed Student's *t*-test.

### Immunofluorescence

Whole heads were embedded and cryo-sectioned along the dorso-ventral plane and fixed in 4% paraformaldehyde and permeabilized with 0.1% Triton X-100 in phosphate buffer (PB). Slides were blocked with 3% bovine serum albumin (BSA) in 0.1 M PB at room temperature for 1 h in a humidified environment. The following primary antibodies were applied overnight at 4°C: anti-GNAT2 (1:400, MBL International, PM075), anti-Zpr3 (1:1000, Zebrafish International Resources Center), anti-EYS (1:300, Novus Biological, NBP1-90038), 1D4 (1:1000, Abcam, AB5417), anti-red (1:300), anti-blue (1:300), anti-green (1:300), and anti-UV (1:300) (kind gifts from Dr. D. Hyde, Zebrafish Research Center, University of Notre Dame, IN, USA). The EYS polyclonal antibody from Novus Biological was raised against a peptide corresponding to EGF domain #11 with a few flanking residues of EGF domain #10 and #12 of human EYS; amino acids 1127-1193 of zebrafish EYS share 53% identity with this antigen (Fig. S1D). After washing with PB containing 0.1% Triton X-100, the slides were incubated with appropriate FITC-conjugated anti-rabbit IgG (1:300, Jackson Immunologicals, 111-095-144) or RITC-conjugated anti-mouse IgG (1:300, Jackson Immunologicals, 115-025-146) for 2 h at room temperature in a humidified environment. Counterstaining with DAPI was used to visualize the nuclei. After washing with PB three times, the sections were covered with VECTASHIELD^®^ mounting medium (Vector Laboratories, Cat# H-1000) by coverslips. To visualize fluorescence, a Zeiss Axioskop epifluorescence microscope and a Zeiss confocal microscope system were used. Epifluorescence images were captured with a mono 12-bit camera and QCapture Pro 6.0 (QImaging). Terminal deoxynucleotidyl transferase dUTP nick end labeling (TUNEL) was carried out with ApopTag Fluorescein *In Situ* Apoptosis Detection Kit (Millipore) according to the manufacturer's suggestions.

For cell counting, two images from the sections with visible nerve heads, one from the dorsal and one from the ventral portion, were captured with a 40× objective at identical locations, halfway between the optic nerve heads and the ciliary margin zone. Images were imported to ImageJ software (NIH) for cell counting. A repeated-measures ANOVA was performed in SPSS (IBM) to determine statistical significance using a 0.05 alpha level. Post-hoc comparisons were done by Student's *t*-test with Bonferroni correction.

### Transmission electron microscopy (EM)

Enucleated eyes were fixed in 2% paraformaldehyde and 2% glutaraldehyde in phosphate buffer. Transmission EM was carried out as we previously described (Liu et al., 2003; Hu et al., 2007).

### 5-Bromo-2′-deoxyuridine (BrdU) labeling

Adult fish were incubated overnight in water containing 5 mM BrdU (Sigma). Whole heads were embedded and cryosectioned along the dorso-ventral plane. Specimen were fixed in 4% PFA for 10 min and pretreated as described (Tang et al., 2007) before immunofluorescence staining. BrdU antibody (1:300, Roche, 11170376001) was applied overnight at 4°C. After washing with PB, slides were incubated with FITC-conjugated anti-mouse IgG (1:300, Jackson Immunoresearch, 115-095-146). The sections were counter-stained with DAPI. Nuclei which were double labeled with DAPI and BrdU at the dorsal ciliary margin, ventral ciliary margin, and central one-third of the retina near the optic nerve head were counted separately.
